# Cyanobacteria as an Experimental Platform for Modifying Bacterial and Plant Photosynthesis

**DOI:** 10.3389/fbioe.2014.00007

**Published:** 2014-04-21

**Authors:** Poul Erik Jensen, Dario Leister

**Affiliations:** ^1^Copenhagen Plant Science Center (CPSC), Department of Plant and Environmental Sciences, University of Copenhagen, Copenhagen, Denmark; ^2^Plant Molecular Biology (Botany), Department of Biology I, Ludwig-Maximilians-University Munich, Munich, Germany

**Keywords:** carboxysome, chloroplast, genetic engineering, photosynthesis, *Synechocystis*, synthetic biology

One of the fascinating characteristics of photosynthesis is its capacity for repair, self-renewal, and energy storage within chemical bonds. Given the evolutionary history of plant photosynthesis and the patchwork nature of many of its components, it is safe to assume that the light reactions of plant photosynthesis can be improved by genetic engineering (Leister, 2012). The evolutionary precursor of chloroplasts was a microorganism whose biochemistry was very similar to that of present-day cyanobacteria. Many cyanobacterial species are easy to manipulate genetically and grow robustly in liquid cultures that can be easily scaled up into photobioreactors. Therefore, cyanobacteria such as *Synechocystis* sp. PCC 6803 (hereafter “*Synechocystis*”) have widely been used for decades as model systems to study the principles of photosynthesis (Table 1). Indeed, genetic engineering based on homologous recombination is well-established in *Synechocystis*. Moreover, new genetic engineering toolkits, including marker-less gene deletion and replacement strategies needing only a single transformation step (Viola et al., 2014) and novel approaches for chromosomal integration and expression of synthetic gene operons (Bentley et al., 2014), allow for large-scale replacement and/or integration of dozens of genes in reasonable time frames. This makes *Synechocystis* a very attractive basis for the experimental modification of important processes like photosynthesis, and it also suggests innovative ways of improving modules of related eukaryotic pathways, among them the combination of cyanobacterial and eukaryotic elements using the tools of synthetic biology.

**Table 1:** **Characteristics of current model systems for photosynthesis**.

Organism	Type of photo-synthesis^a^	Homologous recombination	Life cycle	Shot-gun complementation	Heterotrophic propagation
*Synechocystis*	Prokaryotic	Yes	<1 day	Yes	Yes
*Chlamydomonas*	Eukaryotic	No	<1 day	No	Yes
*Physcomitrella*	Eukaryotic	Yes	Several weeks	No	Restricted^b^
*Arabidopsis*	Eukaryotic	No	Several months	No	Restricted^b^

*^a^This distinction refers to the presence of phycobilisomes (“prokaryotic”) or Lhc proteins (“eukaryotic”) and associated regulatory differences*.

*^b^Refers to non-photoautotrophic mutants*.

## Improving the Photosynthetic Light Reactions in Cyanobacteria

In plants, the activity of the Calvin cycle (in particular the RuBisCO-mediated carbon fixation step) is considered to represent the major brake on photosynthetic efficiency under saturating irradiance and limiting CO_2_ concentrations (Quick et al., 1991; Stitt et al., 1991; Furbank et al., 1996). Autotrophic growth of *Synechocystis*, on the other hand, is constrained by the rate of phosphoglycerate reduction, owing to limitations on the ATP/NADPH supply from the light reactions (Marcus et al., 2011). In fact, cyanobacteria cannot absorb all incoming sunlight due to light reflection, dissipation, and shading effects. In some cases, significant numbers of the photons absorbed by the antennae are not used for energy conversion due to dissipation mechanisms. It has therefore been proposed that uneven light distribution could be avoided by using cell cultures with smaller antenna sizes packed in high-density cell cultures, thus allowing good light penetration into the inner parts of the reactor. Proof of principle for this concept has been obtained in the green alga *Chlamydomonas reinhardtii* (Beckmann et al., 2009), but antenna truncations in *Synechocystis* have so far failed to enhance biomass production (Page et al., 2012). Indeed, increased truncations of phycobilisomes were associated with reductions in photoautotrophic productivity, which were attributed to marked decrease in the PSI:PSII ratio (Collins et al., 2012).

A radically different approach to altering the light-harvesting capability of cyanobacteria and extending the range of wavelengths absorbed involves the introduction into cyanobacteria of the light-harvesting complex II (LHCII) of land plants. In principle, this should be a straightforward exercise, as the complex has a simple structure, containing in its minimal version essentially only one type of Lhcb polypeptide together with chlorophylls (Chl) a and b. Although *Synechocystis* strains that produce large amounts of Chl b in addition to the naturally occurring Chl a have been generated (Xu et al., 2001), the expression of stable Lhcb proteins presents a problem, possibly because they do not fold correctly and are quickly degraded (He et al., 1999). Thus, inefficient light-harvesting remains the principal barrier to high-efficiency *Synechocystis* biomass growth.

## Improving the Photosynthetic Light Reactions of Plants in Cyanobacteria

The gain in photosynthetic efficiency, obtainable when, for instance, photosystems (PS) require less repair and photoprotection, should be significant. It is clear that crop plants and even model plants like *Arabidopsis thaliana* or *Physcomitrella patens* are the systems least suited for testing such approaches, given their long life cycle and inaccessibility to efficient (prokaryote-type) genetic engineering technologies (Table 1). Therefore, redesigning plant PS will require novel model organisms in which such concepts can be implemented, tested, and reiteratively improved. Cyanobacteria, particularly *Synechocystis*, will play an important role in such attempts because of its superior genetic tractability. Thus, the long-term goal is to introduce elements of plant photosynthesis into model cyanobacteria like *Synechocystis* and optimize their effects by genetic engineering. Consequently, chimeric PS employing, for instance, plant cores and antenna complexes from algae could combine features from the whole range of diversity available in eukaryotes, while allowing their impacts to be tested and their properties to be optimized in a prokaryote. Besides the technical advantages of this strategy, it has the added attraction of delegating most of the required work with genetically modified organisms (GMOs) to *Synechocystis*. Reducing the transgenic work done directly in plants might also improve the acceptability of the approach to a public, which has proven to be, at best, skeptical of GMOs.

## Improving CO_2_ Fixation

Cyanobacteria, like plants and algae, use the Calvin cycle for assimilation of CO_2_. The first step in CO_2_ assimilation is the carboxylase reaction catalyzed by RuBisCO, which results in the production of two molecules of 3-phosphoglycerate; one of these is recycled to regenerate ribulose-1,5-bisphosphate (RuBP), whereas the other is converted to biosynthesis of sugars, terpenoids, and fatty acids (Melis, 2013). However, RuBisCO can also react with molecular O_2_ in a process called photorespiration. This oxygenase reaction produces one molecule of 3-phosphoglycerate and one molecule of 2-phosphoglycolate, which acts as an inhibitor of enzymes involved in photosynthetic carbon fixation. Therefore, photorespiration reduces the overall efficiency and output of photosynthesis, since there is a net loss of both CO_2_ and nitrogen.

Of the four distinct forms of RuBisCO (Andersson and Backlund, 2008; Tabita et al., 2008), form I is the most widespread, being found in plants, algae, and cyanobacteria. The cyanobacterial version comprises eight small (RbcS) and eight large (RbcL) subunits. Not surprisingly, RuBisCO is widely conserved across species, but some of its natural variants are slightly more effective than others. For instance, heterologous expression of RuBisCO from the purple-sulfur bacterium *Allochromatium vinosum* in *Synechococcus elongatus* sp. PCC 7942 increased CO_2_ assimilation by almost 50% (Iwaki et al., 2006). Therefore, metagenomic analysis of natural RuBisCO diversity may identify superior enzymes to be engineered into a cyanobacterial host for detailed characterization and platform improvement.

Besides its catalytic subunits RbcL and RbcS, RuBisCO seems to need the molecular chaperone RbcX for proper folding. In some cyanobacteria, the *rbcX* gene co-localizes with the genes encoding RbcL and RbcS in the chromosome. However, to what extent this chaperone is actually needed is still unclear, and the folding/assembly process needs further investigation (for a recent review, see Rosgaard et al., 2012). In plants, activation of RuBisCO by RuBisCO activase is essential for catalysis; however, evidence of a requirement for RuBisCO activase for optimal function of cyanobacterial RuBisCO is lacking (Rosgaard et al., 2012).

Although RuBisCO is the major enzyme responsible for carbon fixation, cyanobacteria possess an additional assimilation mechanism that accounts for nearly 25% of CO_2_ fixation (Yang et al., 2002). Phosphoenolpyruvate carboxylase (PEPC) catalyzes the reaction that fixes HCO_3_^−^ on phosphoenolpyruvate (PEP) to form oxaloacetate and inorganic phosphate in the presence of Mg^2+^ (O’Leary, 1982). This enzyme is widely distributed in all plants and many bacteria. Attempts to improve plant CO_2_ fixation by expression of a cyanobacterial PEPC with diminished sensitivity to feedback inhibition have been unsuccessful; the resulting transgenic plants even showed decreased fitness (Chen et al., 2004).

In the cytosol of cyanobacteria, RuBisCO is found in proteinaceous microcompartments known as carboxysomes (Kerfeld et al., 2010). A carboxysome consists of a shell assembled from roughly 800 protein hexamers, forming the 20 facets of an icosahedron, and 12 pentamers that form its corners (Heinhorst et al., 2006). The carboxysome encapsulates RuBisCO complexes and plays a central role in a mechanism that concentrates inorganic carbon providing enough CO_2_ for the enzyme to favor the carboxylase reaction. In the cytosol, carbonic anhydrases convert CO_2_ to HCO_3_^−^, thereby trapping the inorganic carbon species inside the cells. The carboxysome is rather impermeable to O_2_, but it readily takes up HCO_3_^−^ (Price et al., 2008). Inside the carboxysome, specialized carbonic anhydrases catalyze the release of CO_2_ from the incoming HCO_3_^−^. The number of carboxysomes and the expression levels of carboxysome genes increase significantly when cyanobacterial cells are limited for CO_2_ (Heinhorst et al., 2006). Carboxysomes can potentially be exploited as synthetic compartments, similar to eukaryotic organelles, to rationally organize pathways or networks within a spatially distinct subsystem (Kerfeld et al., 2010).

The terpenoid and fatty acid biosynthetic pathways receive only about 5 and 10% of the photosynthetically fixed carbon, respectively, and this allocation is constitutive but stringently regulated (Melis, 2013). If photosynthetic organisms are to be used as a platform for pathways devoted to the biosynthesis of terpenoid- or fatty acid-derived products, this product-to-biomass carbon portioning must be increased significantly.

## Synthetic Biology

The aim of synthetic biology is to engineer biological systems by designing and constructing novel modules to perform new functions for useful purposes. “Building blocks” (i.e., genes, enzymes, pathways, or regulatory circuits) in synthetic biology are thought of as modular, well-characterized biological parts that can be predictably combined to yield novel and complex cell-based systems following engineering principles (Endy, 2005). In this context, the photosynthetic complexes (PS I and II) in the thylakoids of cyanobacteria can be regarded as building blocks, which can be integrated into novel biosynthetic pathways. Ideally, the biosynthetic pathway should be located in the thylakoids or at least in close proximity to the photosynthetic electron transfer chain, allowing the biosynthetic enzymes to tap directly into photosynthetic electron transport and energy generation, and even draw on carbon skeletons derived from CO_2_ fixation. Recently, an entire cytochrome P450-dependent pathway has been relocated to the thylakoids of tobacco chloroplasts and shown to be driven directly by the reducing power generated by photosynthesis in a light-dependent manner (Zygadlo Nielsen et al., 2013; Lassen et al., 2014). This demonstrates the potential of transferring pathways for structurally complex chemicals to the chloroplast and using photosynthesis to drive the P450s with water as the primary electron donor.

Synthetic biology in cyanobacteria still lags behind conventional species such as *E. coli* and yeast in terms of molecular tools, defined parts, and product yields. Some progress has been made in redirecting photosynthetically fixed carbon toward commercially interesting compounds. The C_5_ molecule isoprene is a volatile hydrocarbon that can be used as fuel and as a platform-chemical for production of synthetic rubber and high-value compounds. For photosynthetic generation of isoprene in cyanobacteria, the isoprene synthase gene from the plant *Pueraria montana* (kudzu) has been successfully expressed in *Synechocystis* and isoprene was indeed produced (Lindberg et al., 2010). However, drastic metabolic engineering will be required to redirect carbon partitioning away from the dominant carbohydrate biosynthesis toward terpenoid biosynthesis. In fact, heterologous expression of the isoprene synthase in combination with the introduction of a non-native mevalonic acid pathway for increased carbon flux toward isopentenyl-diphosphate (IPP) and dimethylallyl-diphosphate (DMAPP) precursors of isoprene resulted in a 2.5-fold improvement in isoprene yield (Bentley et al., 2014).

Tightly regulated and inducible protein expression is an important prerequisite for product yield and predictability in synthetic biology approaches. In this context, riboswitches are attracting increasing interest. Riboswitches are functional non-coding RNA molecules that play a crucial role in gene regulation at the transcriptional or post-transcriptional level in many bacteria (Roth and Breaker, 2009). In general, the sensing domain (aptamer) of riboswitches is combined with a regulating domain. The regulating domain can comprise several types of expression platforms to control gene expression. For instance, direct binding of a specific ligand to the aptamer domain can be used to attenuate transcription termination or translation initiation (Roth and Breaker, 2009). Recently, a theophylline-dependent riboswitch was established as a strict and inducible protein expression system in *S. elongatus* PCC 7942 (Nakahira et al., 2013). Three theophylline riboswitches were tested, and the best one exhibited clear on/off regulation of protein expression. In the ON state, protein expression levels were up to 190-fold higher than in the absence of the activator. Moreover, it was possible to fine-tune the level of protein expression by using a defined range of theophylline concentrations.

## Conclusion

Cyanobacteria are receiving increasing interest as experimental scaffolds for the modification of their endogenous photosynthetic machineries, as well as the integration and engineering of modules of plant photosynthesis. Therefore, we believe that cyanobacteria will be extensively used by many plant biologists as additional model system in future analyses. Indeed, for the identification of the entire set of components necessary for photosynthesis only cyanobacteria are suitable as experimental platforms. If this is achieved, the next goal is to transfer this photosynthetic module to other (non-photosynthetic) organisms like *E. coli*. Moreover, cyanobacteria are attractive as a “green” platform for synthetic biology to produce high-value compounds, chemical feedstocks, or even fuels.

## Conflict of Interest Statement

The authors declare that the research was conducted in the absence of any commercial or financial relationships that could be construed as a potential conflict of interest.

## References

[B1] Andersson I. Backlund A. (2008). Structure and function of Rubisco. Plant Physiol. Biochem. 46, 275–291 10.1016/j.plaphy.2008.01.001 18294858

[B2] Beckmann J. Lehr F. Finazzi G. Hankamer B. Posten C. Wobbe L. (2009). Improvement of light to biomass conversion by de-regulation of light-harvesting protein translation in *Chlamydomonas reinhardtii*. J. Biotechnol. 142, 70–77 10.1016/j.jbiotec.2009.02.015 19480949

[B3] Bentley F. K. Zurbriggen A. Melis A. (2014). Heterologous expression of the mevalonic acid pathway in cyanobacteria enhances endogenous carbon partitioning to isoprene. Mol. Plant 7, 71–86 10.1093/mp/sst134 24157609

[B4] Chen L. M. Li K. Z. Miwa T. Izui K. (2004). Overexpression of a cyanobacterial phosphoenolpyruvate carboxylase with diminished sensitivity to feedback inhibition in *Arabidopsis* changes amino acid metabolism. Planta 219, 440–449 10.1007/s00425-004-1244-3 15054659

[B5] Collins A. M. Liberton M. Jones H. D. Garcia O. F. Pakrasi H. B. Timlin J. A. (2012). Photosynthetic pigment localization and thylakoid membrane morphology are altered in *Synechocystis* 6803 phycobilisome mutants. Plant Physiol. 158, 1600–1609 10.1104/pp.111.192849 22331410 PMC3320172

[B6] Endy D. (2005). Foundations for engineering biology. Nature 438, 449–453 10.1038/nature04342 16306983

[B7] Furbank R. T. Chitty J. A. Von Caemmerer S. Jenkins C. (1996). Antisense RNA inhibition of RbcS gene expression reduces Rubisco level and photosynthesis in the C4 Plant *Flaveria bidentis*. Plant Physiol. 111, 725–734 12226324 10.1104/pp.111.3.725 PMC157888

[B8] He Q. Schlich T. Paulsen H. Vermaas W. (1999). Expression of a higher plant light-harvesting chlorophyll a/b-binding protein in *Synechocystis* sp. PCC 6803. Eur. J. Biochem. 263, 561–570 10.1046/j.1432-1327.1999.00526.x 10406967

[B9] Heinhorst S. Cannon G. C. Shively J. M. (2006). “Carboxysomes and carboxysome-like inclusions,” in Complex Intracellular Structures in Prokaryotes, ed. Shively J. M. (Heidelberg: Springer), 141–164

[B10] Iwaki T. Haranoh K. Inoue N. Kojima K. Satoh R. Nishino T. (2006). Expression of foreign type I ribulose-1,5-bisphosphate carboxylase/oxygenase (EC 4.1.1.39) stimulates photosynthesis in cyanobacterium *Synechococcus* PCC7942 cells. Photosyn. Res. 88, 287–297 10.1007/s11120-006-9048-x 16741604

[B11] Kerfeld C. A. Heinhorst S. Cannon G. C. (2010). Bacterial microcompartments. Annu. Rev. Microbiol. 64, 391–408 10.1146/annurev.micro.112408.134211 20825353

[B12] Lassen L. M. Zygadlo Nielsen A. Ziersen B. Gnanasekaran T. Møller B. L. Jensen P. E. (2014). Redirecting photosynthetic electron flow into light-driven synthesis of alternative products including high-value bioactive natural compounds. ACS Synth. Biol. 3, 1–12 24328185 10.1021/sb400136f

[B13] Leister D. (2012). How can the light reactions of photosynthesis be improved in plants? Front. Plant Sci. 3:199 10.3389/fpls.2012.00199 22973282 PMC3428562

[B14] Lindberg P. Park S. Melis A. (2010). Engineering a platform for photosynthetic isoprene production in cyanobacteria, using *Synechocystis* as the model organism. Metab. Eng. 12, 70–79 10.1016/j.ymben.2009.10.001 19833224

[B15] Marcus Y. Altman-Gueta H. Wolff Y. Gurevitz M. (2011). Rubisco mutagenesis provides new insight into limitations on photosynthesis and growth in *Synechocystis* PCC6803. J. Exp. Bot. 62, 4173–4182 10.1093/jxb/err116 21551078 PMC3153676

[B16] Melis A. (2013). Carbon partitioning in photosynthesis. Curr. Opin. Chem. Biol. 17, 453–456 10.1016/j.cbpa.2013.03.010 23542013

[B17] Nakahira Y. Ogawa A. Asano H. Oyama T. Tozawa Y. (2013). Theophylline-dependent riboswitch as a novel genetic tool for strict regulation of protein expression in cyanobacterium *Synechococcus elongatus* PCC 7942. Plant Cell Physiol. 54, 1724–1735 10.1093/pcp/pct115 23969558

[B18] O’Leary M. H. (1982). Phosphoenolpyruvate carboxylase: an enzymologist’s view. Annu. Rev. Plant Physiol. 33, 297–315 10.1146/annurev.pp.33.060182.001501

[B19] Page L. E. Liberton M. Pakrasi H. B. (2012). Reduction of photoautotrophic productivity in the cyanobacterium *Synechocystis* sp. strain PCC 6803 by phycobilisome antenna truncation. Appl. Environ. Microbiol. 78, 6349–6351 10.1128/AEM.00499-12 22706065 PMC3416626

[B20] Price G. D. Badger M. R. Woodger F. J. Long B. M. (2008). Advances in understanding the cyanobacterial CO_2_-concentrating-mechanism (CCM): functional components, Ci transporters, diversity, genetic regulation and prospects for engineering into plants. J. Exp. Bot. 59, 1441–1461 10.1093/jxb/erm112 17578868

[B21] Quick W. P. Schurr U. Scheibe R. Schulze E. D. Rodermel S. R. Bogorad L. (1991). Decreased ribulose-1,5-bisphosphate carboxylase-oxygenase in transgenic tobacco transformed with “antisense” *rbcS*: I. Impact on photosynthesis in ambient growth conditions. Planta 183, 542–554 10.1007/BF00194276 24193848

[B22] Rosgaard L. De Porcellinis A. J. Jacobsen J. H. Frigaard N. U. Sakuragi Y. (2012). Bioengineering of carbon fixation, biofuels, and biochemicals in cyanobacteria and plants. J. Biotechnol. 162, 134–147 10.1016/j.jbiotec.2012.05.006 22677697

[B23] Roth A. Breaker R. R. (2009). The structural and functional diversity of metabolite-binding riboswitches. Annu. Rev. Biochem. 78, 305–334 10.1146/annurev.biochem.78.070507.135656 19298181 PMC5325118

[B24] Stitt M. Quick W. P. Schurr U. Schulze E. D. Rodermel S. R. Bogorad L. (1991). Decreased ribulose-1,5-bisphosphate carboxylase-oxygenase in transgenic tobacco transformed with ‘antisense’ rbcS: II. Flux-control coefficients for photosynthesis in varying light, CO_2_, and air humidity. Planta 183, 555–566 10.1007/BF00194277 24193849

[B25] Tabita F. R. Satagopan S. Hanson T. E. Kreel N. E. Scott S. S. (2008). Distinct form I, II, III, and IV Rubisco proteins from the three kingdoms of life provide clues about Rubisco evolution and structure/function relationships. J. Exp. Bot. 59, 1515–1524 10.1093/jxb/erm361 18281717

[B26] Viola S. Rühle T. Leister D. (2014). A single vector-based strategy for marker-less gene replacement in *Synechocystis* sp. PCC 6803. Microb. Cell Fact. 13, 4 10.1186/1475-2859-13-4 24401024 PMC3893515

[B27] Xu H. Vavilin D. Vermaas W. (2001). Chlorophyll b can serve as the major pigment in functional photosystem II complexes of cyanobacteria. Proc. Natl. Acad. Sci. U.S.A. 98, 14168–14173 10.1073/pnas.251530298 11717469 PMC61186

[B28] Yang C. Hua Q. Shimizu K. (2002). Metabolic flux analysis in synechocystis using isotope distribution from ^13^C-labeled glucose. Metab. Eng. 4, 202–216 12616690 10.1006/mben.2002.0226

[B29] Zygadlo Nielsen A. Ziersen B.E. Jensen K. Lassen L.M. Olsen C.E. Møller B.L. (2013). Redirecting photosynthetic reducing power towards bioactive natural product synthesis. ACS Synth. Biol. 2, 308–315 23654276 10.1021/sb300128r

